# Transcranial Doppler Sonography Reveals Reductions in Hemispheric Asymmetry in Healthy Older Adults during Vigilance

**DOI:** 10.3389/fnagi.2017.00021

**Published:** 2017-02-08

**Authors:** Amanda E. Harwood, Pamela M. Greenwood, Tyler H. Shaw

**Affiliations:** ARCH Laboratory, Department of Psychology, George Mason UniversityFairfax, VA, USA

**Keywords:** transcranial doppler, vigilance, cognitive aging, cognitive resources, compensatory effort

## Abstract

Given that older adults are remaining longer in the workforce, their ability to perform demanding cognitive tasks such as vigilance assignments needs to be thoroughly examined, especially since many vigilance assignments affect public safety (e.g., aviation, medicine and long distance driving). Previous research exploring the relation between aging and vigilance is conflicted, with some studies finding decreased vigilance performance in older adults but others finding no effect of age. We sought a better understanding of effects of age on vigilance by assessing neurophysiological change over the course of a vigil in young (aged 18–24) and healthy older (aged 66–77) adults. To measure temporal changes in cerebral blood flow, participants underwent functional transcranial doppler (fTCD) recording during a 1 h vigilance task. Based on research showing a compensatory effect of increased left hemisphere activation during vigilance in young adults and the “hemispheric asymmetry reduction in older adults” (HAROLD) model, we predicted that during vigilance our older adults would show greater left hemisphere activation but perform at a similar level compared to young adults. While cerebral blood flow velocity (CBFV) declined over time in both groups, only young adults showed the typical right-lateralized CBFV pattern. Older adults showed greater left hemisphere activation consistent with the HAROLD model. However, the increased left hemisphere activation did not appear to be compensatory as the older adults performed at a significantly lower level compared to young adults over the vigil. Findings are discussed in terms of the HAROLD model of healthy aging and the resource theory of vigilance.

## Introduction

Older people are now remaining longer in the workforce than has been the case historically. It is projected that over 30% of people aged 65–74 will still be working in 2022, up from 20% in 2002 (Toossi, 2012). In light of well-documented age-related declines in fluid cognitive ability (Park et al., 2002), this change raises questions about risks to public safety. This may be particularly relevant for jobs with high cognitive demand, such as jobs requiring vigilance or sustained attention. Many real-life vigilance assignments affect public safety such as aviation (i.e., air traffic control, aircraft cockpit monitoring, TSA baggage inspection), military (i.e., intelligence gathering, cyber-security, seaboard navigation, unmanned vehicle flight), homeland security, medicine and long distance driving (Parasuraman, 1979; Warm et al., 2008; Shaw et al., 2009; Helton et al., 2010). Vigilance performance is known to decline over time, a phenomenon known as the vigilance decrement. The decrement is evident as early as 5 min into the vigil (Teichner, 1974; Warm et al., 2008) though more typically within the first 15–30 min (e.g., Mackworth, 1948). Despite this rapid decrement, many real-life vigilance assignments last for several hours with few rest breaks. While some professions requiring vigilance specifically exclude workers over a certain age (e.g., air traffic control), other professions do not (e.g., anesthesiology). Therefore, it is important to assess vigilance performance in retirement-aged people that are now in the workforce in increasing numbers. Our approach to a better understanding of effects of aging on vigilance was to measure the neurophysiology underlying vigilance performance in older and younger adults using functional transcranial doppler sonography (fTCD).

Despite considerable research, there is no consensus concerning the effects of healthy aging on the vigilance decrement (e.g., Davies and Parasuraman, 1982; McAvinue et al., 2012). Some studies find that older adults show poorer vigilance performance than young adults (Filley and Cullum, 1994; Mouloua and Parasuraman, 1995; Berardi et al., 2001; McAvinue et al., 2012), while others find no age-related difference (Parasuraman and Davies, 1977; Davies and Parasuraman, 1982; Parasuraman et al., 1989; Parasuraman and Giambra, 1991; Deaton and Parasuraman, 1998). For example, in a large longitudinal study with multiple age cohorts from 18 to 80+, Giambra and Quilter (1988) found no effect of age on vigilance performance. In another large study, McAvinue et al. (2012) did find effects of age on vigilance performance on the Sustained Attention to Response Task (SART; Robertson et al., 1997). They found that younger adults outperformed their older counterparts. An examination of the underlying neurophysiology of aging during vigilance may shed light on the underlying performance processes involved, which could help to resolve some of the inconsistency in the literature.

Although there have been several theories of the vigilance decrement, there are currently two predominant theories: mindlessness theory and resource theory (Wickens, 2002; Warm et al., 2008; Helton et al., 2010). Mindlessness theory suggests that poor performance on vigilance arises when participants withdraw attention from the task due to the monotonous—yet easy—nature of the task (Robertson et al., 1997; Helton et al., 2005). In contrast, resource theory suggests that the vigilance decrement can be better attributed to the participant expending attentional resources from a limited pool that is depleted with continuous task performance (Wickens, 2002; Warm et al., 2008; Helton et al., 2010). While there is evidence to support both theories, the resource theory of vigilance is more comprehensively supported by studies using various methods of measuring workload including self-report measures—such as NASA-TLX (Hart and Staveland, 1988) or the Multiple Resource Questionnaire (MRQ; Boles and Adair, 2001). By understanding the perceived workload of a task through such self-report workload measures, decrements in performance can be attributed to work and effort during a vigil rather than task disengagement (Hitchcock et al., 2003; Warm and Parasuraman, 2009; Shaw et al., 2010, 2013a; Finomore et al., 2013).

Perhaps the strongest evidence for resource theory comes from neurophysiological studies of vigilance (Parasuraman et al., 1998; Lim et al., 2010; Langner et al., 2012; Shaw et al., 2013b). While several neurophysiological tools have been used to study brain activity during vigilance, fTCD has shown a notably consistent pattern of results. fTCD is an ultrasound procedure that allows for continuous monitoring of cerebral blood flow velocity (CBFV) in the main-stem intracranial arteries. The logic underlying fTCD is that when a particular area of the brain becomes metabolically active, such as during the performance of mental tasks, there is an increase in by-products of metabolic activity such as carbon dioxide (CO_2_). The increase in CO_2_ leads to an increase in oxygenated blood flow velocity to that region to remove the waste products (Aaslid, 1986). The middle cerebral artery (MCA) is commonly used to measure CBFV for complex cognitive tasks as it supplies 80% of the blood to each respective cerebral hemisphere’s frontal lobe (Stroobant and Vingerhoets, 2000; Warm et al., 2008).

Resource theory is also supported by findings that the absolute level of blood flow velocity in the brain varies directly with task difficulty (Hitchcock et al., 2003; Warm and Parasuraman, 2009; Shaw et al., 2010, 2012, 2013a), and the vigilance decrement is paralleled by a temporal decline in cerebral hemovelocity (Shaw et al., 2009; Warm and Parasuraman, 2009). Importantly, these CBFV effects are not present in control observers who are exposed to an identical vigilance task with no work imperative (Hitchcock et al., 2003; Shaw et al., 2009, 2012). In addition, the CBFV effects are lateralized to the right cerebral hemisphere, consistent with PET and fMRI studies that point to a right-hemispheric system in the functional control of vigilance performance (Parasuraman et al., 1998; Langner and Eickhoff, 2013). However, much of the recent research has revealed that vigilance tasks imposing a strong demand on observers require resource recruitment from both the left and right cerebral hemispheres (Helton et al., 2010; Shaw et al., 2012, 2016). Thus, bilateral increases in CBFV may be indicative of higher effort.

In light of evidence that bilateral hemispheric activation may help to maintain vigilance performance in young people (Shaw et al., 2016), the motivating question for the current work was whether bilateral activation would also benefit vigilance performance in older people. Previous research has shown that for tasks during which young people show largely unilateral Blood Oxygen Level Dependent (BOLD) signals, healthy older people show bilateral activation. This has been seen in tasks of episodic memory, semantic memory, working memory, perception and inhibitory control (Grady et al., 1994, 2005) and confirmed in meta-analyses (Spreng et al., 2010; Maillet and Rajah, 2014). Cabeza (2002) synthesized this research into the Hemispheric Asymmetry Reduction in Older Adults (HAROLD) hypothesis of functional brain organization. Cabeza (2002) speculated that older adults use bilateral activation in the prefrontal cortex either to compensate for cognitive decline or as a result of what Baltes and Lindenberger (1997) termed dedifferentiation indicating age-related reductions in brain specialization. Monitoring CBFV during a vigilance task may provide insight into this question by determining whether older adults show an increase in overall CBFV or whether the fTCD measure reveals reduced hemispheric asymmetry during vigilance.

The current investigation used fTCD to examine the neurophysiological correlates in healthy old age during the performance of a vigilance task. While previous research on this subject is mixed, we predicted that young adults would outperform older adults on measures of vigilance (i.e., higher hit rate, lower false alarm rate and faster reaction time). However, we predicted that this performance difference would be reduced if older adults showed more bilateral activation as revealed by CBFV. Thus, under the HAROLD model and the resource model of vigilance, we predicted greater bilateral activation in older compared to young adults during vigilance.

## Materials and Methods

### Participants

Thirty participants were recruited into the study (Table 1). Young adults consisted of 15 participants (age range 18–24, *M* = 20 years) and were recruited from a large university in the Mid-Atlantic region of the United States. These participants were given course credit for their participation. Older adults consisted of 15 participants (age range 66–77, *M* = 69) and were recruited from local “lifelong learning” organizations as well as from fliers posted in the area surrounding the university. Older participants were given travel compensation of up to 10 dollars. Participants in both groups were all right-handed as indicated by self-report. Exclusion criteria included current use of psychoactive medications, recent (<6 months) concussion, and/or cardiovascular disorders (i.e., history of stroke). All participants underwent a brief cognitive assessment at the end of the experiment. Table 1 shows average demographic information and the mean scores on the cognitive assessment tests. It should be noted that the lowest individual Mini-Mental State Examination (MMSE) score was 26, above the cutoff used by the Alzheimer’s Disease Neuroimaging Initiative (ADNI). The study was approved by the Universities’ Institutional Review Board.

**Table 1:** **Group demographic and cognitive assessment information**.

Group	*n*	Gender	Age	MMSE	WMSi	WMSd	Flanker Inc-Cong_1_	CVS slope_2_
Young adults	15	4 Males	20	N/A	8.75_3_	6.25_3_	−64.40	5.528
Older adults	15	7 Males	69	29	11.75	10.00	−94.41	6.133

*^1^Eriksen flanker variables were analyzed using the compatibility effect—the average reaction time of the incongruent trials (Inc) minus the average reaction time of the congruent trials (Cong). ^2^Cued visual search (CVS) slope refers to the slope of the Cue Size/RT function. ^3^One young adult (non-native English speaker) was excluded from the WMSi and WMSd calculation due to low performance on those tests only*.

### Materials and Measures

#### Mini-Mental State Examination

Older participants were administered the MMSE (Folstein et al., 1975), a standard screening tool to eliminate volunteers who may be suffering from a dementing disease. The cut-off criterion was set at 26 out of a possible 30 points; no volunteers were excluded from participation based on the MMSE.

#### Vigilance Task

**Figure 1:**
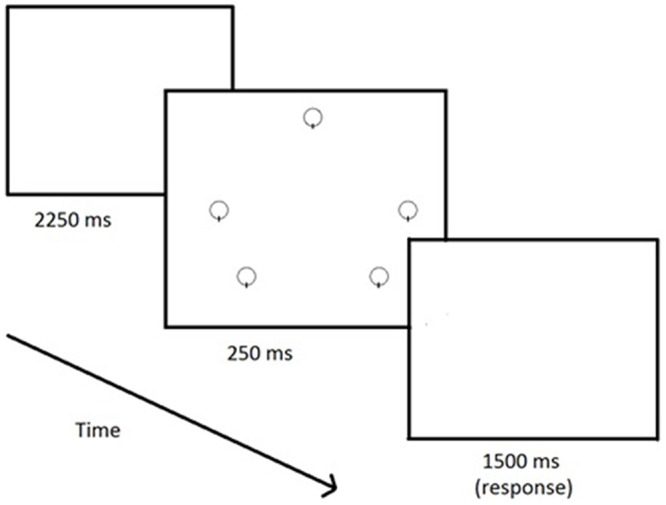
**Graphical depiction of a neutral trial**.

Participants participated in a continuous vigilance task during which they viewed the repetitive presentation of an array of five open circles (14 mm diameter) outlined by a 1 mm black line that appeared on a white background on a 17-inch computer screen. The circles were positioned 75 mm from the center of the screen at the 3, 5, 7, 9 and 12 o’clock locations. The critical signal for detection was the absence of a vertical 1 mm black line intersecting the 6 o’clock position within *one* of the five circles in the display. This display was chosen because of previous research that has shown that it exhibits appropriate psychometric properties and is of sufficient difficulty to influence performance (Finomore et al., 2006; Shaw et al., 2016). An example of the stimulus array is presented in the center panel in Figure 1. Prior to engaging in the actual vigilance testing session, all participants were given a brief practice session. The practice consisted of 75 trials, five critical (one for each position) and 70 neutral with auditory performance feedback (i.e., “hit”, “miss”, or “false”). The experimental test session consisted of six blocks of 150 trials, 10 critical (2 per position) and 140 neutral, without auditory feedback. Each trial consisted of a blank screen (2250 ms), followed by the stimulus (250 ms, with neutral or critical), a blank response screen (1500 ms) for a total of 4 s per trial. The entire vigilance task lasted for 60 min.

Events were presented serially, and the display was updated once every 4 s, resulting in an event rate of 15 events/min. All stimuli were exposed for 0.25 s. For each observer, critical signals came into view on an average of once per minute during each period of watch (signal probability = 0.067) and the signals appeared equally often on each of the five circles comprising the vigilance display. Participants signified their detection of critical signals by pressing the space bar on a computer keyboard. Responses occurring within 1.5 s after the appearance of critical signals were recorded as correct detections; responses to non-signal events were classified as false alarms.

#### Wechsler Memory Scale

The Wechsler Memory Scale (WMS Form 1, Wechsler, 1997) is a standard neuropsychological assessment of immediate and delayed episodic memory. The WMS, Logical Memory subtest, was administered by having the researcher first read the standard instructions for the test, and then read a paragraph aloud to the participant. Participants were asked to immediately recall the story aloud (WMS-Immediate score). After 30 min of another task and without being told in advance that they would be asked again to recall, the participants were asked again to recall as much of the story as they could (WMS-Delayed score). The researcher followed standardized instructions to score the test.

#### Eriksen Flanker

In the Eriksen Flanker task (EF; Eriksen and Eriksen, 1974) the participants were presented with a set of five arrows “<<<<<”. Participants were required to indicate if the center arrow is facing right “>” or left “>” using the keyboard (“A” for left, “L” for right). There were two types of trials: congruent and incongruent. For the congruent trials, all the arrows pointed the same direction as the target arrow “<<<<<” or “>>>>>”. In the incongruent trials, the other four arrows pointed the opposite directions as the target arrow “<<><<” or ‘>><>>”. The participants were instructed to respond as quickly and as accurately as possible throughout the 15-min task. Data were pre-processed as compatibility effect reaction times (calculated using: [average incongruent trial reaction time] − [average congruent trial reaction time]; Lavie and Cox, 1997).

#### Cued Visual Search

For the cued visual search (CVS) task, participants monitored the computer screen for the presence of a pink “T” in a three by five array of letters. In most trials, the array of letters was preceded by a cue, which was a box drawn around one letter, one column, three adjacent columns, or the entire array. The participants were instructed to make a speeded response by pressing the spacebar to indicate when they saw the target. The target was present during 50% of the 1-min practice and 83.33% of the experimental trials. The entire task lasted 15 min. Data were calculated using the slope of the Cue Size/RT function (Greenwood and Parasuraman, 1999).

### Procedure

Participants were greeted and shown to a windowless laboratory. After a short verbal description of the experiment, participants read and signed the consent form, and filled out a biographical questionnaire. Older participants were then administered the MMSE. Participants were then fitted with the fTCD sensors.

Prior to the vigilance practice session, participants were linked to a fTCD unit (Spencer Technologies model PMD150). The unit is equipped with two 2 MHz pulsed transducers embedded in a plastic bracket that is secured around the head with an adjustable Velcro strap. Both the left and right transducers were placed along the temporal bone dorsal and immediately proximal to the zygomatic arch. Ultrasound transmission gel was placed between each TCD transducer and the participant’s skin to obtain a clear ultrasound signal. CBFV was measured from the mainstream MCA for the left and right cerebral hemispheres and provided a reading in cm/s. The MCA was monitored at approximate depths of 50–55 mm. The MCA is the artery most often measured in vigilance studies, as it supplies about 80% of the blood to the brain (Stroobant and Vingerhoets, 2000). A baseline was acquired by having the participant stare at a blank screen for 5 min. Blood flow data for the last 60 s of the baseline period was used as the baseline index, consistent with previous research (Aaslid, 1986; Shaw et al., 2009). Subjects wore the TCD headset for the duration of the experiment.

Next, the participant completed a short practice of the main vigilance task. In order to be retained in the vigilance experiment, the participant was required to have scored 80% or higher on hits and commit no more than 10% false alarms. If necessary, participants completed a second practice. Two participants required a second practice, while five participants were unable to meet this inclusion criterion and were therefore excluded from the experiment. Participants that did meet the practice criterion went on to complete the test trials of the vigilance task which lasted 60 min. After completion, the fTCD sensors were removed and the participant was given a 5-min break prior to the cognitive assessment.

For the cognitive assessment, the participant was first administered the WMSi (~3 min). Next, they completed the EF task (~ 15 min) and the CVS task (~15 min), in counterbalanced order. The participant was then read the recall directions of the delayed WMS (WMSd). Finally, the participant was thanked for his or her participation and the researcher provided a synopsis of the goals of the study and answered any questions. The younger participants were provided course credit and the older participants were offered ten dollars for travel compensation. The entire duration of the experiment was approximately 120 min. Data for the vigilance task and CBFV were examined using mixed-model analysis of variance (ANOVAs). Data for the Cognitive Assessment were analyzed using a MANOVA.

## Results

### Cognitive Assessment

Table 1 shows that older adults on average scored 29 out of 30 on the MMSE (the lowest score was 26). A MANOVA conducted on the EF, CVS, WMSi and WMSd revealed a significant difference (Wilks lambda; *F*_(3,25)_ = 4.666, *p* < 0.01, *η*^2^ = 0.427). Follow-up univariate analyses revealed that older adults had higher rates of immediate and delayed recall in the WMSi (*F*_(1,28)_ = 6.288, *p* < 0.05, *η*^2^ = 0.183) and WMSd (*F*_(1,28)_ = 7.570, *p* = 0.01, *η*^2^ = 0.183) memory tests. However, older adults had more interference due to incongruent stimuli as seen in the reaction time (when subtracting out the individual difference of reaction time for congruent stimuli) on the EF test (*F*_(1,28)_ = 5.375, *p* < 0.05, *η*^2^ = 0.161). While not significant, older adults had slightly higher slopes on the CVS cue size/reaction time function. Although the older adults were slightly slower to respond, they were not cognitive impaired.

### Vigilance Performance

**Figure 2:**
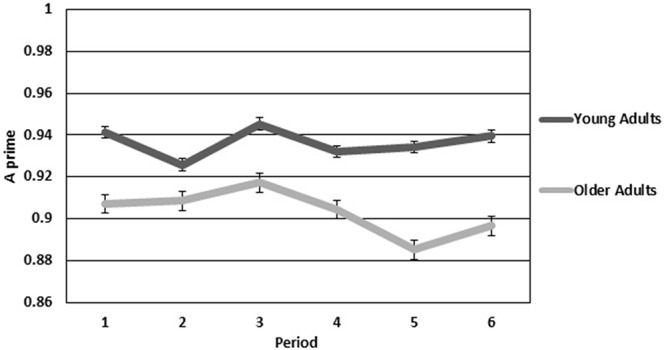
**Sensitivity for older and younger adults over the six continuous 10-min periods of the vigil**. Error bars are standard error.

In all repeated measures analyses, violations of sphericity were corrected using the Greenhouse-Geisser correction. Performance on the vigilance task was examined using the A prime statistic (Pollack and Norman, 1964), a nonparametric signal detection measure that is used to look at perceptual sensitivity (a ratio of correct detections to false alarms). Figure 2 shows A prime means for each period plotted for both older and younger adults. A mixed-model two (age group) by six (period of watch) ANOVA revealed a significant main effect for group (*F*_(1,28)_ = 5.508, *p* < 0.05, *η*^2^ = 0.164), such that younger adults had higher perceptual sensitivity than older adults. Neither the main effect of period or the interaction of group by period were significant.

**Figure 3:**
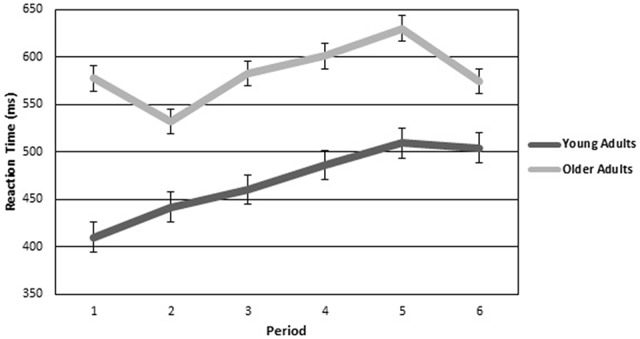
**Reaction Time for older and younger adults over the six continuous 10-min periods of the vigil**. Error bars are standard error.

Reaction time to the target was also investigated with a two (age group) by six (period of watch) ANOVA. The analysis revealed a significant main effect of period (*F*_(5,98.692)_ = 3.472, *p* < 0.05, *η*^2^ = 0.126), such that reaction time increased with time. Figure 3 is a graphical representation of reaction time for each period plotted for both older and younger adults. There was a marginal main effect of group (*F*_(1,24)_ = 3.948, *p* = 0.058) such that older adults were slower to respond than young adults. There was no significant interaction.

### Cerebral Blood Flow Velocity

**Figure 4:**
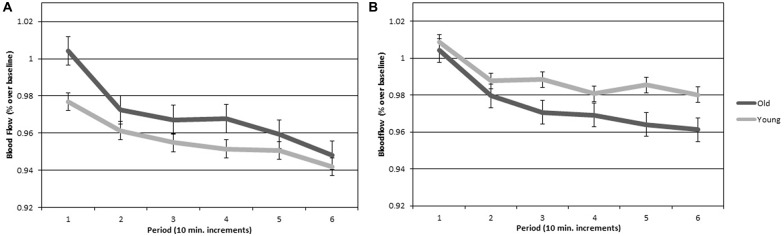
**(A)** Cerebral hemovelocity relative to baseline (percent) plotted for older and younger adults for the left cerebral hemisphere over the six continuous 10-min periods of the vigil. **(B)** Cerebral hemovelocity relative to baseline (percent) plotted for older and younger adults for the right cerebral hemisphere over the six continuous 10-min periods of the vigil. Error bars are standard error.

CBFV scores for all participants were expressed as a proportion of the last minute of their 5-min baseline, consistent with previous work (Warm and Parasuraman, 2007; Shaw et al., 2016). Figure 4 plots CBFV over time for left and right hemispheres for each age group. Importantly, there was no significant difference in baseline scores between groups (*p* > 0.05) on neither the left (young *M* = 57.86, SEM = 2.13; old *M* = 55.62, SEM = 1.93) or right (young *M* = 58.13, SEM = 2.10; old *M* = 56.27, SEM = 1.94) hemispheres. Thus, differences in hemovelocity during the vigil cannot be attributed to differences at baseline. A two (hemisphere) by two (age group) by six (periods of watch) ANOVA was conducted on the CBFV scores. There was a significant main effect of hemisphere (*F*_(1,28)_ = 7.205, *p* < 0.05, *η*^2^ = 0.206), such that CBFV was higher in the right hemisphere than the left. There was also a significant main effect of period (*F*_(1,84.661)_ = 7.205, *p* < 0.01, *η*^2^ = 0.292), such that CBFV declined over time. There was not a significant main effect of group. There was a significant hemisphere by group interaction (*F*_(1,27)_ = 7.205, *p* < 0.05, *η*^2^ = 0.211). There were no other significant main effects or interactions.

**Figure 5:**
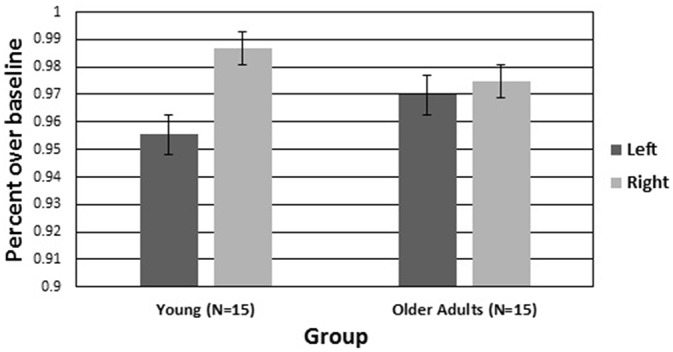
**Average cerebral hemovelocity relative to baseline (percent) plotted for older and younger adults for left and right cerebral hemispheres over the 60-min vigil**. Error bars are standard error.

The hemisphere by group interaction for CBFV was followed up with a simple effects analysis comparing hemisphere for each group. There was a significant difference between the left and right hemispheres in young adults with higher CBFV in the right hemisphere (*F*_(1,27)_ = 7.205, *p* < 0.05, *η*^2^ = 0.228). There was not a significant difference between the left and right hemispheres in older adults. Figure 5 is a visual representation of CBFV for left and right cerebral hemispheres plotted for both older and younger adults. It can be seen in the figure that hemispheric asymmetry is reduced in older adults. To test the hypotheses that increased bilateral activation would have a compensatory effect on vigilance performance in older adults, a Pearson correlation analyses was conducted on the mean A prime scores and mean difference score between right and left hemisphere CBFV. The correlation was not significant (*p* > 0.05).

## Discussion

To test our hypothesis that having more resources committed to a vigilance task heightens performance regardless of age, we used CBFV to measure resources during vigilance in young and older people. Previous work has shown that task-related brain activation is more bilateral in healthy older people compared to young people. This bilateral activation has been linked to a compensatory mechanism for poorer cognitive performance (reviewed in Greenwood, 2007). Based on evidence that: (a) in young people vigilance performance is improved in groups who activate CBFV bilaterally (Shaw et al., 2016); and (b) high- but not low-functioning older people showed a bilateral activation pattern (Cabeza et al., 2002), we predicted that healthy older people would experience a benefit in vigilance performance from bilateral activation. Consistent with previous findings (Rogers, 2000; Warm et al., 2008), we found the young group showed stronger right than left hemisphere activation during vigilance. Although the healthy, cognitively normal (Table 1) older group showed bilateral activation, they also showed poorer vigilance performance than the young adults. These findings are relevant to several questions.

First, we found that older people did not perform as well as young on vigilance tasks. While the literature does not consistently show that vigilance is age sensitive (reviewed in Giambra, 1997), our study is consistent with such a conclusion. This is an important matter in light of the growing presence of retirement-aged people in the workforce, including in vigilance assignments relevant to public safety.

Second, our findings are relevant to the debate on whether increased regional activation in older people is related to a compensatory process. Beginning with the work of Grady et al. (1994, 2005), it has been found that tasks that induce unilateral hemispheric activation in young people instead induce bilateral activation in older people. Such evidence is the basis for the HAROLD hypothesis which argues that the decreased lateralization seen in older adults reflects either compensation or dedifferentiation (Cabeza, 2002). Greenwood (2007) hypothesized that in aging, functional plasticity leads to changed processing strategy which increases activation of atrophic regions (prefrontal and parietal) and thereby improves performance. Consistent with that, there is recent evidence of “compensation by recruitment” in older people in memory processes (Lighthall et al., 2014; Kennedy et al., 2015; Fernández-Cabello et al., 2016), with confirmation by meta-analyses (Spreng et al., 2010; Maillet and Rajah, 2014). Although we did observe increased activation of frontal regions among the older people, that bilateral activation occurred together with poorer vigilance performance. Therefore, if the increased activation of the left hemisphere was compensatory, that compensation was not sufficient to raise the performance of the healthy old to the level of the young. Thus, our results from a vigilance task do not support the idea of “compensation by recruitment” in aging. However, there is previous evidence that compensation by recruitment occurs in young extraverts during vigilance performance (Shaw et al., 2016). Our findings that the older group showed more bilateral activation but poorer performance relative to the young group is consistent with dedifferentiation theory (Baltes and Lindenberger, 1997). That view predicts that age-related loss of regional brain specificity reflected in bilateral processing is accompanied by poorer performance. The hypothesis of dedifferentiation does not predict compensation. However, more recent hypotheses of brain aging have predicted it. First, Greenwood (2007) argued from the paradoxically increased activation of brain regions known to shrink with age that age-related reductions in regional brain integrity drives both changes in processing strategy and functional cortical reorganization. Subsequently, Park and Reuter-Lorenz (2009) advanced a very similar view, termed “scaffolding theory.” The present study did not find evidence that the observed bilateral activation in the older group had compensatory effects. Considering the existing evidence together, it appears that compensation by recruitment can occur in both young and older people. Nevertheless, our results showed that for vigilance tasks, bilateral recruitment may be relatively ineffective in ameliorating the performance of older people.

Third, our findings add to the literature reporting variability in the strength of the vigilance decrement. We did not see a traditional vigilance decrement in performance sensitivity. One explanation could have been the relatively slow event rate (15 events/min). Event rate has been found to be a primary determinant of the vigilance decrement, such that slow event rates (e.g., 5 events/min) are not as resource demanding as faster event rates (30 events/min; Parasuraman and Davies, 1977). Indeed, the performance difference between older and younger adults was restricted to an overall difference in perceptual sensitivity. It is worth noting, though, that a significant slowing of reaction time was observed in the study, and there was a marginally significant main effect that points to more slowing in the older group. So, while both groups were able to maintain their detection performance over time, both older and younger adults slowed in their response over the course of the vigil, which could be evidence of the decrement function.

Our findings raise the question of whether, in light of public safety concerns, age alone is a sufficient predictor of vigilance performance in the real world. In a study that compared high- and low-cognitive functioning older adults, only the high-functioning group showed bilateral activation pattern (Cabeza et al., 2002). This suggests the importance of understanding the effect of overall cognitive functioning on the ability to compensate by recruitment. It will be important for future research to compare higher- and lower-functioning older adults for the ability to compensate for cognitive decline. A study of this sort is especially relevant given the relatively recent findings relating to increased “brain reserve” among older adults, in which older adults that engage in demanding cognitive and motor activities seem to show strengthened brain machinery (Freret et al., 2015).

It will also be important for future research to consider other sources of individual differences in vigilance performance and neurophysiological response. Shaw and colleagues (Shaw et al., 2010; Matthews et al., 2011, 2014) have shown the importance of linking operator characteristics to neurophysiological measures to gain insight into how individual differences affect resource allocation strategies. Recent research in this vein has considered a wide variety of operator characteristics such as extraversion (Shaw et al., 2010, 2016), neuroticism (Mandell et al., 2015), impulsivity (Shaw et al., 2010) and experience (Shaw et al., 2013b). This work reveals individual differences in both vigilance performance and hemispheric activation patterns. Although this suggests there may be some ability to compensate to maintain vigilance, the effect may be fragile or dependent upon a combination of multiple factors that were not considered in this study.

## Ethics Statement

This study was carried out in accordance with the recommendations of Human Subjects SOPs, Office of Research Integrity and Assurance’s Institutional Review Board with written informed consent from all subjects. All subjects gave written informed consent in accordance with the Declaration of Helsinki. The protocol was approved by the Office of Research Integrity and Assurance’s Institutional Review Board.

## Author Contributions

The study was conceived and designed by THS, PMG and AEH. Data collection and analyses were conducted by AEH. The article was written and revised by AEH, THS and PMG.

## Funding

Supported by Air Force Office of Scientific Research (AFOSR/AFRL) Grant FA9550-10-1-0385 and the Center of Excellence in Neuroergonomics, Technology and Cognition (CENTEC). Publication of this article was funded in part by the George Mason University Libraries Open Access Publishing Fund.

## Conflict of Interest Statement

The authors declare that the research was conducted in the absence of any commercial or financial relationships that could be construed as a potential conflict of interest.

## References

[B1] Aaslid R. Ed. (1986). “Transcranial Doppler examination techniques,” in Transcranial Doppler Sonography, (Vienna: Springer), 39–59.

[B2] Baltes P. B. Lindenberger U. (1997). Emergence of a powerful connection between sensory and cognitive functions across the adult life span: a new window to the study of cognitive aging? Psychol. Aging 12, 12–21. 10.1037/0882-7974.12.1.12 9100264

[B3] Berardi A. Parasuraman R. Haxby J. (2001). Overall vigilance and sustained attention decrements in healthy aging. Exp. Aging Res. 27, 19–39. 10.1080/03610730126014 11205528

[B4] Boles D. B. Adair L. P. (2001). “The multiple resources questionnaire (MRQ),” in Proceedings of the Human Factors and Ergonomics Society 45th Annual Meeting, (Vol. 45, No. 25), (Thousand Oaks, CA: SAGE Publications), 1790–1794.

[B5] Cabeza R. (2002). Hemispheric asymmetry reduction in older adults: the HAROLD model. Psychol. Aging 17, 85–100. 10.1037/0882-7974.17.1.85 11931290

[B6] Cabeza R. Anderson N. D. Locantore J. K. McIntosh A. R. (2002). Aging gracefully: compensatory brain activity in high-performing older adults. Neuroimage 17, 1394–1402. 10.1006/nimg.2002.1280 12414279

[B7] Davies D. R. Parasuraman R. (1982). The Psychology of Vigilance. San Diego, CA: Academic Press.

[B8] Deaton R. Parasuraman R. (1998). “Age differences in sensory and cognitive vigilance using tactical symbolic displays,” in Human Cognition: A Multidisciplinary Perspective, eds Singh I. L. Parasuraman R. (New Delhi: Sage Publications), 165–183.

[B9] Eriksen B. A. Eriksen C. W. (1974). Effects of noise letters upon identification of a target letter in a nonsearch task. Percept. Psychophys. 16, 143–149. 10.3758/bf03203267

[B10] Fernández-Cabello S. Valls-Pedret C. Schurz M. Vidal-Piñeiro D. Sala-Llonch R. Bargallo N.. (2016). White matter hyperintensities and cognitive reserve during a working memory task: a functional magnetic resonance imaging study in cognitively normal older adults. Neurobiol. Aging 48, 23–33. 10.1016/j.neurobiolaging.2016.08.008 27636672

[B11] Filley C. M. Cullum C. M. (1994). Attention and vigilance functions in normal aging. Appl. Neuropsychol. 1, 29–32. 10.1207/s15324826an01012_6 16318558

[B12] Finomore V. S. Jr. Shaw T. H. Warm J. S. Matthews G. Boles D. B. (2013). Viewing the workload of vigilance through the lenses of the NASA-TLX and the MRQ. Hum. Factors 55, 1044–1063. 10.1177/0018720813484498 24745198

[B13] Finomore V. S. Warm J. S. Matthews G. Riley M. A. Dember W. N. Shaw T. H. (2006). “Measuring the workload of sustained attention,” in Proceedings of the Human Factors and Ergonomics Society 50th Annual Meeting, (Santa Monica, CA: Human Factors and Ergonomics Society), 1614–1618.

[B14] Folstein M. F. Folstein S. E. McHugh P. R. (1975). “Mini-mental state”. A practical method for grading the cognitive state of patients for the clinician. J. Psychiatr. Res. 12, 189–198. 10.1016/0022-3956(75)90026-6 1202204

[B15] Freret T. Gaudreau P. Schumann-Bard P. Billard J. M. Popa-Wagner A. (2015). Mechanisms underlying the neuroprotective effect of brain reserve against late life depression. J. Neural Transm. (Veinna) 122, S55–S61. 10.1007/s00702-013-1154-2 24390152

[B16] Giambra L. M. (1997). Sustained attention and aging: overcoming the decrement? Exp. Aging Res. 23, 145–161. 10.1080/03610739708254030 9151075

[B17] Giambra L. M. Quilter R. E. (1988). Sustained attention in adulthood: a unique, large-sample, longitudinal and multicohort analysis using the Mackworth Clock-Test. Psychol. Aging 3, 75–83. 10.1037/0882-7974.3.1.75 3268245

[B18] Grady C. L. Maisog J. M. Horwitz B. Ungerleider L. G. Mentis M. Salerno J. A.. (1994). Age-related processing changes in cortical blood flow activation of faces and location during visual. J. Neurosci. 14, 1450–1462. 8126548 10.1523/JNEUROSCI.14-03-01450.1994 PMC6577560

[B19] Grady C. L. McIntosh A. R. Craik F. I. M. (2005). Task-related activity in prefrontal cortex and its relation to recognition memory performance in young and old adults. Neuropsychologia 43, 1466–1481. 10.1016/j.neuropsychologia.2004.12.016 15989937

[B20] Greenwood P. M. (2007). Functional plasticity in cognitive aging: review and hypothesis. Neuropsychology 21, 657–673. 10.1037/0894-4105.21.6.657 17983277

[B21] Greenwood P. M. Parasuraman R. (1999). Scale of attentional focus in visual search. Percept. Psychophys. 61, 837–859. 10.3758/bf03206901 10498999

[B22] Hart S. G. Staveland L. E. (1988). Development of NASA-TLX (Task Load Index): results of empirical and theoretical research. Adv. Psychol. 52, 139–183. 10.1016/s0166-4115(08)62386-9

[B23] Helton W. S. Hollander T. D. Warm J. S. Matthews G. Dember W. N. Wallaart M.. (2005). Signal regularity and the mindlessness model of vigilance. Br. J. Psychol. 96, 249–261. 10.1348/000712605x38369 15969834

[B24] Helton W. S. Warm J. S. Tripp L. D. Matthews G. Parasuraman R. Hancock P. A. (2010). Cerebral lateralization of vigilance: a function of task difficulty. Neuropsychologia 48, 1683–1688. 10.1016/j.neuropsychologia.2010.02.014 20171235

[B25] Hitchcock E. M. Warm J. S. Matthews G. Dember W. N. Shear P. K. Tripp L. D. (2003). Automation cueing modulates cerebral blood flow and vigilance in a simulated air traffic control task. Theor. Issues Ergon. Sci. 4, 89–112. 10.1080/14639220210159726

[B26] Kennedy K. Rodrigue K. Bischof G. N. Hebrank A. Reuter-Lorenz P. Park D. C. (2015). Age trajectories of functional activation under conditions of low and high processing demands: an adult lifespan fmri study of the aging brain. Neuroimage 104, 21–34. 10.1016/j.neuroimage.2014.09.056 25284304 PMC4252495

[B27] Langner R. Eickhoff S. B. (2013). Sustaining attention to simple tasks: a meta-analytic review of the neural mechanisms of vigilant attention. Psychol. Bull. 139, 870–900. 10.1037/a0030694 23163491 PMC3627747

[B28] Langner R. Kellermann T. Eickhoff S. B. Boers F. Chatterjee A. Willmes K.. (2012). Staying responsive to the world: modality-specific and-nonspecific contributions to speeded auditory, tactile and visual stimulus detection. Hum. Brain Mapp. 33, 398–418. 10.1002/hbm.21220 21438078 PMC6870397

[B29] Lavie N. Cox S. (1997). On the efficiency of visual selective attention: efficient visual search leads to ineffective distractor rejection. Psychol. Sci. 8, 395–398. 10.1111/j.1467-9280.1997.tb00432.x

[B30] Lighthall N. R. Huettel S. A. Cabeza R. Carolina N. (2014). Functional compensation in the ventromedial prefrontal cortex improves memory-dependent decisions in older adults. J. Neurosci. 34, 15648–15657. 10.1523/JNEUROSCI.2888-14.2014 25411493 PMC4236396

[B31] Lim J. Wu W. C. Wang J. Detre J. A. Dinges D. F. Rao H. (2010). Imaging brain fatigue from sustained mental workload: an ASL perfusion study of the time-on-task effect. Neuroimage 49, 3426–3435. 10.1016/j.neuroimage.2009.11.020 19925871 PMC2830749

[B32] Mackworth N. H. (1948). The breakdown of vigilance during prolonged visual search. Q. J. Exp. Psychol. 1, 6–21. 10.1080/17470214808416738

[B33] Maillet D. Rajah M. N. (2014). Age-related differences in brain activity in the subsequent memory paradigm: a meta-analysis. Neurosci. Biobehav. Rev. 45, 246–257. 10.1016/j.neubiorev.2014.06.006 24973756

[B34] Mandell A. R. Becker A. VanAndel A. Nelson A. Shaw T. H. (2015). Neuroticism and vigilance revisited: a transcranial doppler investigation. Conscious. Cogn. 36, 19–26. 10.1016/j.concog.2015.05.017 26057404

[B35] Matthews G. Warm J. S. Reinerman-Jones L. E. Langheim L. K. Guznov S. Shaw T. H. (2011). The functional fidelity of individual differences research: the case for context-matching. Theor. Issues Ergon. Sci. 12, 435–450. 10.1080/1463922x.2010.549247

[B36] Matthews G. Warm J. S. Shaw T. H. Finomore V. S. (2014). Predicting battlefield vigilance: a multivariate approach to assessment of attentional resources. Ergonomics 57, 856–875. 10.1080/00140139.2014.899630 24678837

[B37] McAvinue L. P. Habekost T. Johnson K. A. Kyllingsbæk S. Vangkilde S. Bundesen C.. (2012). Sustained attention, attentional selectivity, and attentional capacity across the lifespan. Atten. Percept. Psychophys. 74, 1570–1582. 10.3758/s13414-012-0352-6 22825931

[B38] Mouloua M. Parasuraman R. (1995). Aging and cognitive vigilance: effects of spatial uncertainty and event rate. Exp. Aging Res. 21, 17–32. 10.1080/03610739508254265 7744168

[B39] Parasuraman R. (1979). Memory load and event rate control sensitivity decrements in sustained attention. Science 205, 924–927. 10.1126/science.472714 472714

[B41] Parasuraman R. Davies D. R. (1977). “A taxonomic analysis of vigilance,” in Vigilance: Theory, Operational Performance and Physiological Correlates, ed. Mackie R. R. (New York, NY: Plenum Press), 559–574.

[B40] Parasuraman R. Giambra L. (1991). Skill development in vigilance: effects of event rate and age. Psychol. Aging 6, 155–169. 10.1037/0882-7974.6.2.155 1863385

[B42] Parasuraman R. Nestor P. Greenwood P. (1989). Sustained-attention capacity in young and older adults. Psychol. Aging 4, 339–345. 10.1037/0882-7974.4.3.339 2803627

[B43] Parasuraman R. Warm J. S. See J. E. (1998). “Brain systems of vigilance,” in The Attentive Brain, ed. Parasuraman R. (Cambridge, MA: MIT Press), 221–256.

[B45] Park D. C. Lautenschlager G. Hedden T. Davidson N. S. Smith A. D. Smith P. K. (2002). Models of visuospatial and verbal memory across the adult life span. Psychol. Aging 17, 299–320. 10.1037/0882-7974.17.2.299 12061414

[B44] Park D. C. Reuter-Lorenz P. (2009). The adaptive brain: aging and neurocognitive scaffolding. Annu. Rev. Psychol. 60, 173–196. 10.1146/annurev.psych.59.103006.093656 19035823 PMC3359129

[B46] Pollack I. Norman D. A. (1964). A non-parametric analysis of recognition experiments. Psychon. Sci. 1, 125–126. 10.3758/bf03342823

[B47] Robertson I. H. Manly T. Andrade J. Baddeley B. T. Yiend J. (1997). ’Oops!’: performance correlates of everyday attentional failures in traumatic brain injured and normal subjects. Neuropsychologia 35, 747–758. 10.1016/s0028-3932(97)00015-8 9204482

[B48] Rogers L. J. (2000). Evolution of hemispheric specialization: advantages and disadvantages. Brain Lang. 73, 236–253. 10.1006/brln.2000.2305 10856176

[B49] Shaw T. H. Finomore V. S. Warm J. S. Matthews G. (2012). Effects of regular or irregular event schedules on cerebral hemovelocity during a sustained attention task. J. Clin. Exp. Neuropsychol. 34, 57–66. 10.1080/13803395.2011.621890 22053921

[B50] Shaw T. H. Funke M. E. Dillard M. Funke G. J. Warm J. S. Parasuraman R. (2013a). Event-related cerebral hemodynamics reveals target-specific resource allocation for both “go” and “no-go” response-based vigilance tasks. Brain Cogn. 82, 265–273. 10.1016/j.bandc.2013.05.003 23727665

[B53] Shaw T. H. Satterfield K. Ramirez R. Finomore V. (2013b). Using cerebral hemovelocity to measure workload during a spatialized auditory vigilance task for novice and experienced observers. Ergonomics 56, 1251–1263. 10.1080/00140139.2013.809154 23789766

[B51] Shaw T. H. Matthews G. Warm J. S. Finomore V. S. Silverman L. Costa P. T. (2010). Individual differences in vigilance: personality, ability and states of stress. J. Res. Pers. 44, 297–308. 10.1016/j.jrp.2010.02.007

[B52] Shaw T. H. Nguyen C. Satterfield K. Ramirez R. McKnight P. E. (2016). Cerebral hemovelocity reveals differential resource allocation strategies for extraverts and introverts during vigilance. Exp. Brain Res. 234, 577–585. 10.1007/s00221-015-4481-8 26563163

[B54] Shaw T. H. Warm J. S. Finomore V. Tripp L. Matthews G. Weiler E.. (2009). Effects of sensory modality on cerebral blood flow velocity during vigilance. Neurosci. Lett. 461, 207–211. 10.1016/j.neulet.2009.06.008 19539707

[B55] Spreng R. N. Wojtowicz M. Grady C. L. (2010). Reliable differences in brain activity between young and old adults: a quantitative meta-analysis across multiple cognitive domains. Neurosci. Biobehav. Rev. 34, 1178–1194. 10.1016/j.neubiorev.2010.01.009 20109489

[B56] Stroobant N. Vingerhoets G. (2000). Transcranial Doppler ultrasonography monitoring of cerebral hemodynamics during performance of cognitive tasks: a review. Neuropsychol. Rev. 10, 213–231. 10.1023/A:1026412811036 11132101

[B57] Teichner W. H. (1974). The detection of a simple visual signal as a function of time of watch. Hum. Factors 16, 339–353. 10.1177/001872087401600402 4435787

[B58] Toossi M. (2012). Labor force projections to 2020: a more slowly growing workforce, (January), 2010–2020. Available online at: http://www.bls.gov/opub/mlr/2012/01/art3full.pdf

[B59] Warm J. S. Parasuraman R. (2007). “Cerebral hemodynamics and vigilance,” in Neuroergonomics: The Brain at Work, eds Parasuraman R. Rizzo M. (New York, NY: Oxford University Press), 146–158.

[B60] Warm J. S. Parasuraman R. (2009). “Cerebral hemodynamics and vigilance,” in Neuroergonomics: The Brain at Work, 75–100.

[B61] Warm J. S. Parasuraman R. Matthews G. (2008). Vigilance requires hard mental work and is stressful. Hum. Factors 50, 433–441. 10.1518/001872008X312152 18689050

[B62] Wechsler D. (1997). Wechsler Memory Scale. 3rd Edn. San Antonio, TX: Psychological Corporation.

[B63] Wickens C. D. (2002). Multiple resources and performance prediction. Theor. Issues Ergon. Sci. 3, 159–177. 10.1080/14639220210123806

